# Learning shapes the aversion and reward responses of lateral habenula neurons

**DOI:** 10.7554/eLife.23045

**Published:** 2017-05-31

**Authors:** Daqing Wang, Yi Li, Qiru Feng, Qingchun Guo, Jingfeng Zhou, Minmin Luo

**Affiliations:** 1 School of Life Sciences, Tsinghua University, Beijing, China; 2 National Institute of Biological Sciences, Beijing, China; Columbia University in the City of New York, United States

**Keywords:** punishment, social defeat, Pavlovian conditioning, reward, single-unit recordings, fiber photometry, Mouse

## Abstract

The lateral habenula (LHb) is believed to encode negative motivational values. It remains unknown how LHb neurons respond to various stressors and how learning shapes their responses. Here, we used fiber-photometry and electrophysiology to track LHb neuronal activity in freely-behaving mice. Bitterness, pain, and social attack by aggressors intensively excite LHb neurons. Aversive Pavlovian conditioning induced activation by the aversion-predicting cue in a few trials. The experience of social defeat also conditioned excitatory responses to previously neutral social stimuli. In contrast, fiber photometry and single-unit recordings revealed that sucrose reward inhibited LHb neurons and often produced excitatory rebound. It required prolonged conditioning and high reward probability to induce inhibition by reward-predicting cues. Therefore, LHb neurons can bidirectionally process a diverse array of aversive and reward signals. Importantly, their responses are dynamically shaped by learning, suggesting that the LHb participates in experience-dependent selection of behavioral responses to stressors and rewards.

**DOI:** http://dx.doi.org/10.7554/eLife.23045.001

## Introduction

‘Making profits and avoiding loss’ is the alpha rule for an organism’s survival. Surviving in a challenging environment requires animals to select appropriate behaviors to avoid danger. The lateral habenula (LHb) in the epithalamus plays a critical role in processing aversive signals (Hikosaka, 2010; Proulx et al., 2014). The LHb participates in processing behavioral responses to pain, anxiety, reward, and stress (Katz et al., 1981; Dafny and Qiao, 1990; Haun et al., 1992; Wirtshafter et al., 1994; Sharp et al., 2006; Clark et al., 2009). Activating LHb inputs from the basal ganglia or the LHb outputs to the midbrain monoaminergic centers results in avoidance and ‘fear-like’ behaviors (Lammel et al., 2012; Shabel et al., 2012; Stamatakis and Stuber, 2012; Yang et al., 2016). Bilaterally ablating the LHb impairs avoidance learning (Wilcox et al., 1986). Pharmacological inactivation of LHb neurons makes animals indifferent to the cost of a given behavioral outcome (Stopper and Floresco, 2014). Moreover, dysfunctions of the LHb are associated with several devastating mental disorders, including depression, schizophrenia, and drug withdrawal symptoms (Morris et al., 1999; Shumake et al., 2003; Shepard et al., 2006; Yang et al., 2008; Salas et al., 2009; Li et al., 2011, Li et al., 2013; Tost et al., 2015).

Electrophysiological recordings have revealed several major insights into how LHb neurons contribute to the processing of aversive signals. In the primate LHb, many neurons are strongly inhibited by reward-predicting events and strongly excited by disappointment (*i.e.*, the failure of obtaining an expected reward) (Matsumoto and Hikosaka, 2007, 2009). A subpopulation of LHb neurons was shown to be activated by aversive airpuff treatment to the face or a neutral sensory cue that predicts the delivery of an airpuff (Matsumoto and Hikosaka, 2009). These studies indicate that the LHb provides negative motivational value signals, such as the absence of a reward or the presence of a punishment, to inhibit downstream dopaminergic neurons and serotonergic neurons in the midbrain (Varga et al., 2003; Matsumoto and Hikosaka, 2007; Hikosaka, 2010; Shabel et al., 2012; Stamatakis et al., 2013; Root et al., 2014). This attractive hypothesis is supported by recent results showing that habenular lesion reduces the inhibitory responses of dopamine neurons to reward omission (Tian and Uchida, 2015).

In the present study, we aimed to address how LHb neurons in freely-behaving animals dynamically encode aversive or reward signals during the learning process. Considering that a majority of studies that use electrophysiological recording to study aversion have used airpuffs as the aversive stimulus, we are of the view that it is important to test additional aversive stimuli, such as bitter tastants, pain, and social punishments, to examine whether LHb neurons respond generally to punishment signals. Moreover, previous electrophysiological recording studies have focused on well-trained monkeys. It remains unclear how the learning process shapes the response patterns of LHb neurons. Here, we examined the response profiles of LHb neurons by combining fiber photometry and single-unit electrophysiological recordings in freely-behaving mice. Our results indicate that stressors in general strongly excite LHb neurons. We also found that a sucrose reward evokes a response pattern that consists of an initial inhibition followed by excitation in many LHb neurons. Recordings from mice engaged in Pavlovian conditioning tasks further revealed that LHb neurons, through learning, rapidly gain excitatory responses to punishment-predicting cues but develop inhibitory responses to reward-predicting cues much more slowly. Our findings suggest that the LHb may participate in bidirectional anticipation and tracking the negative values of stressors and the positive values of rewards.

## Results

### Bitter taste, pain, and social stressors activate LHb neurons

**Figure 1.**
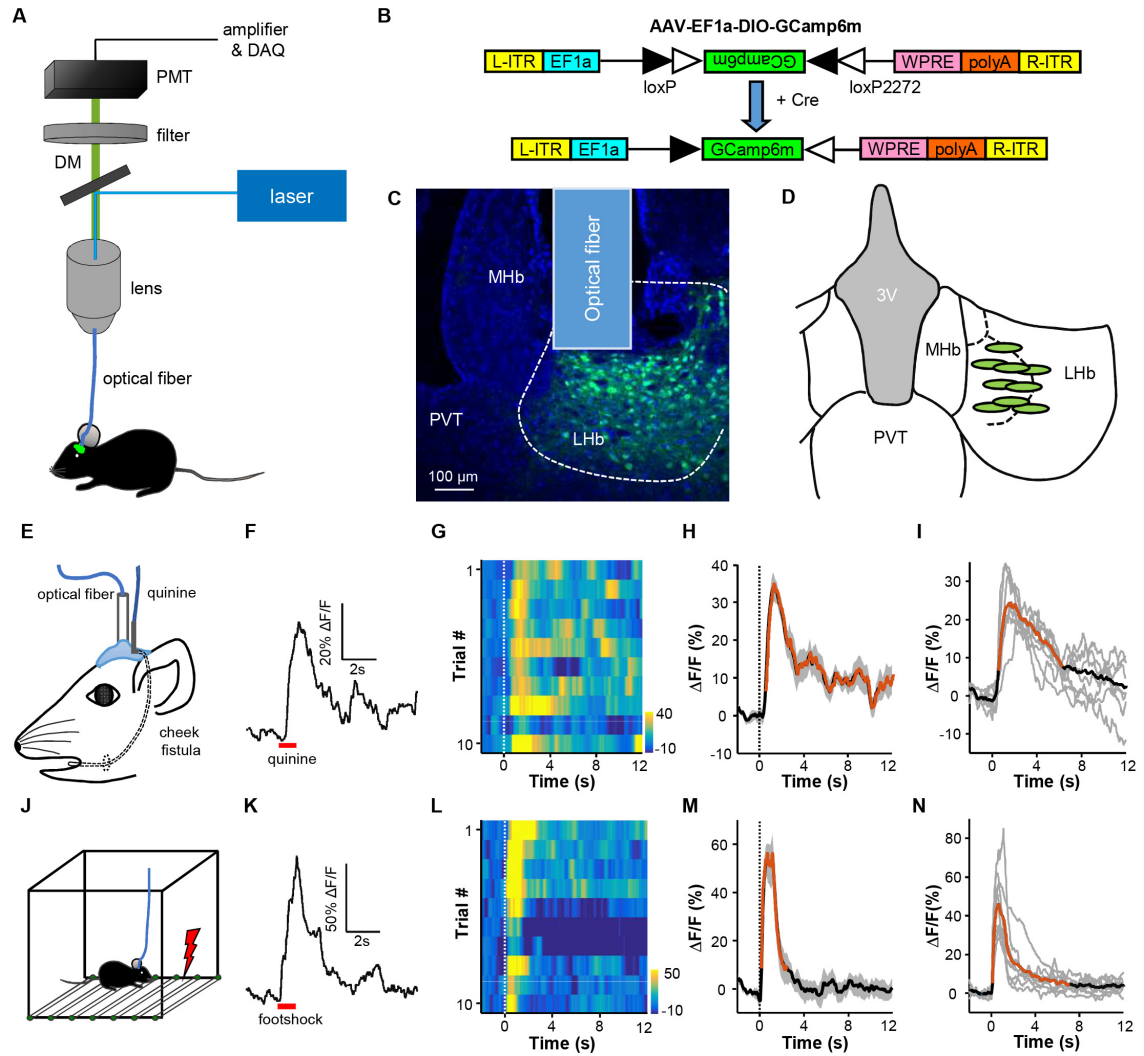
Intra-oral quinine delivery and footshock increase Ca^2+^ signals in VGlut2-expressing neurons in the LHb. (**A**) Schematic of the fiber-photometry setup. We recorded Ca^2+^ transients from GCaMP6-expressing neurons from the LHb of freely behaving mice. DM, dichroic mirror; PMT, photomultiplier tube. (**B** and **C**) Injecting recombinant AAV-DIO-GCaMP6m (**B**) into the LHb of a *Slc17a6-ires-Cre* (Vglut2-LHb-GCaMP6) mouse resulted in GCaMP6m expression (green) in LHb neurons (**C**). Postmortem examination verified the location of the implanted optical fiber. Blue, DAPI counterstaining of cell nuclei. (**D**) Recording sites within the LHb (n = 9 mice). Each green dot represents the center of optical tip in an individual mouse. (**E**) The schematics of intra-oral solution infusion through a cheek fistula. (**F**) Raw trace of fluorescence changes shows that intra-oral delivery of quinine (horizontal bar) rapidly increased GCaMP6 signals within one test trial. (**G** and **H**) Trial-by-trial heatmap representation of GCaMP6m transients evoked by random quinine infusion (n = 10 trials; **G**) and peri-event plot of the average Ca^2+^ transient for a mouse (**H**). Color scale indicates the range of *ΔF/F* in (**G**); (**I**) Average Ca^2+^ signals associated with intra-oral quinine infusion for the entire test group (n = 7 mice). (**J–N**) The effects of footshock. (**J**) Schematics showing footshock application. (**K**) The raw trace shows a footshock-evoked change in GCaMP6 fluorescence within one test trial. (**L**) Heatmap representation of GCaMP6 signals across trials. (**M**) Average GCaMP6 transients across trials for the same mouse shown in (**C**). (**N**) Average GCaMP6 transients for the entire test group (n = 8 mice). In (H, I, M, and N), thick lines indicate the mean, shaded areas indicate the SEM, and the dashed lines indicate the onset of quinine infusion or footshock. Red segments indicate statistically-significant increases from the baseline (p<0.05; multivariate permutation tests). **DOI:** http://dx.doi.org/10.7554/eLife.23045.002

We used fiber photometry to record Ca^2+^ transients as the real-time activity indicator for LHb neurons in freely-behaving mice (Adelsberger et al., 2005; Cui et al., 2013; Gunaydin et al., 2014)(Figure 1A). A vast majority of neurons in the LHb are glutamatergic and express the marker vesicular glutamate transporter 2 (Vglut2; encoded by the gene *Slc17a6*; Aizawa et al., 2012). We expressed the genetically encoded Ca^2+^ indicator, GCaMP6m, in LHb neurons by stereotaxically infusing the Cre-dependent adeno-associated virus AAV-DIO-GCaMP6m into the LHb of the *Slc17a6-ires-Cre mice* (henceforth referred to as Vglut2-LHb-GCaMP6 mice) (Figure 1B; Figure 1C). A small optical fiber was implanted into the LHb to record the changes in GCaMP6 fluorescence (Figure 1C; Figure 1D).

**Figure 1—figure supplement 1.**
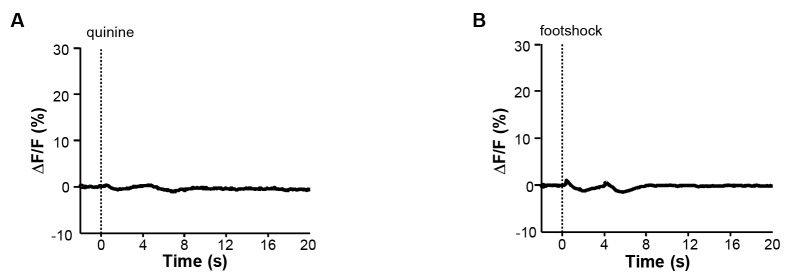
Quinine and footshock do not evoke substantial changes in GFP fluorescence in the control Vglut2-LHb-EmGFP mice. (**A**) Effect of intra-oral quinine infusion (dashed vertical line) on the GFP fluorescence in the LHb of Vglut2-LHb-EmGFP mice (n = 5 mice). (**B**) Effect of footshock on the GFP fluorescence in the LHb of control mice (n = 5 mice). **DOI:** http://dx.doi.org/10.7554/eLife.23045.003

We first examined how intra-oral infusion of quinine affected the GCaMP signals of LHb neurons. Randomly delivering a small amount of quinine into the mouse oral cavity (5% w/v, 10 μL in 0.5s) reliably evoked Ca^2+^ transients across trials for an individual mouse (Figure 1E–1H), resulting in a significant increase of Ca^2+^ signals in all of the tested mice (Figure 1I; n = 7 mice; 27.8 ± 2.4% *ΔF/F* mean ± SEM). The signals rose rapidly from the quinine onset (time to peak 0.68 ± 0.14 s mean ± SEM) and decayed slowly following quinine offset (decay time constant 5.88 ± 0.73 s). Intra-oral quinine infusion did not produce any change in fluorescence levels from the LHb of EmGFP-expressing control mice (Figure 1—figure supplement 1A), indicating that the GCaMP signals were indeed derived from quinine-evoked changes in cellular Ca^2+^ levels but not movement-related artifacts. These recordings thus indicate that bitter taste strongly activates LHb neurons.

Footshock, a painful stimulus, similarly evoked strong Ca^2+^ transients from LHb neurons (Figure 1J). Random footshocks (0.6 mA, 0.5 s) rapidly induced an intensive increase of GCaMP signals (Figure 1K). This increase was reliably detected across 10 test trials of an animal (Figure 1L; Figure 1M). For all mice tested, the GCaMP signals were strong (48.8 ± 7.3% *ΔF/F*) and fast (time to peak 0.26 ± 0.04 s and decay time 1.89 ± 0.47 s; Figure 1N; n = 8 mice). We noted that the decay time for quinine was longer than that for footshock, likely due to slow clearance of quinine from the oral cavity. Again, we did not observe any clear fluorescence changes following footshocks for control EmGFP-expressing mice (Figure 1—figure supplement 1B). Therefore, pain rapidly and strongly activates LHb neurons.

**Figure 2.**
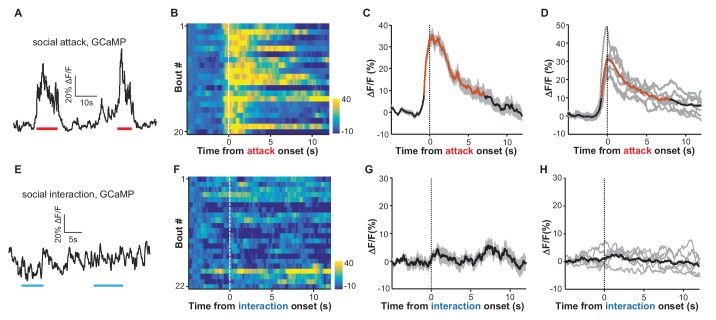
Social attack by an aggressor activates LHb neurons. (**A–D**) Ca^2+^ signals in the LHb neurons of test male mice increased when an aggressive resident male attacked the test male. A Vglut2-LHb-GCaMP6 male mouse was introduced into the home cage of a CD-1 male mouse (aggressor), who often initiated attacks on the test male. GCaMP fluorescence changes were segmented and aligned to the onset of attack by the resident male. (**A**) Raw trace of GCaMP6m fluorescence levels in response to social defeat. (**B**) Heatmap representation of Ca^2+^ transients in the LHb neurons of a Vglut2-LHb-GCaMP6 mouse. Each row represents a bout. (**C**) Peri-event plot of the average Ca^2+^ transients from the same mouse shown in (**B**). (**D**) Mean Ca^2+^ signals for the entire test group (n = 7 Vglut2-LHb-GCaMP6 mice). In (**C** and **D**), thick lines indicate the mean and shaded areas indicate the SEM. Red segments indicate statistically-significant increases from the baseline (p<0.05; multivariate permutation test). (**E–H**) Raw trace (**E**), heatmap (**F**), and average Ca^2+^ transient of an individual mouse (**G**), as well as the average plot for the entire test group (**H**), all showing the lack of clear Ca^2+^ signals during the social interaction between the test male and its non-aggressive male littermate (n = 6 Vglut2-LHb-GCaMP6 mice). Same conventions as in (**A–D**). **DOI:** http://dx.doi.org/10.7554/eLife.23045.004

We next investigated how LHb neurons responded to social stressors. To induce acute social stress, we introduced a test mouse into the home cage of an aggressive CD-1 male mouse and simultaneously videoed the fighting episodes and monitored the Ca^2+^ signals from the LHb of the test mouse. After smelling and chasing the test mouse for a few minutes, the aggressive CD-1 intruder mouse typically started attacking the test mouse, who in turn tried to retreat to escape the attack. The Ca^2+^ signals increased when the test mouse was chased and peaked immediately when it was attacked (Figure 2A and Video 1). Aligning the Ca^2+^ signals according to attack onset revealed reliable increases in LHb neuronal activity across fighting bouts (Figure 2B; Figure 2C). Multivariate permutation tests for the entire group of test mice revealed a significant elevation of Ca^2+^ signals during social aggression interactions (Figure 2D; 32.9 ± 3.5% *ΔF/F* mean ± SEM; time to peak 0.57 ± 0.08 s; decay time constant 7.80 ± 0.76 s). These increases could not have been caused by movement-related artifacts, as we did not observe any significant changes in the EmGFP fluorescence in EmGFP-expressing mice that were subjected to similar social aggression (Figure 2—figure supplement 1A–D). Importantly, social aggression, rather than general social interaction (with a nonaggressive littermate), activated LHb neurons. This was revealed by recording test mice that were investigating and interacting with a non-aggressive male littermate. The test male often initiated chemoinvestigation following the introduction of a non-aggressive male into its home cage. We did not observe a reliable change in Ca^2+^ signals from any of the test mice involved in such social interactions (Figure 2E–2H).

**Figure 2—figure supplement 1.**
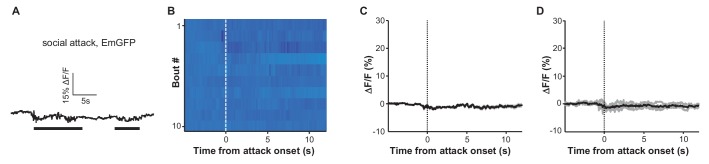
Social attack by an aggressor does not produce any clear change in green fluorescence in the EmGFP-expressing control mice. (**A**) Raw trace. (**B**) Heatmap representation of 10 fighting bouts. (**C**) Average GFP fluorescence change of the same mouse as shown in (**B**). (**D**) Mean fluorescence change of the test population (n = 4 Vglut2-LHb-EmGFP control mice). **DOI:** http://dx.doi.org/10.7554/eLife.23045.005

**Video 1.** Social attack by an aggressor activates LHb neurons. Using fiber photometry, we recorded GCaMP signals from the LHb neurons of a male mouse. Following its introduction to the cage of an aggressive CD-1 male, this test animal was repetitively attacked by the CD-1 aggressor. The social attack was reliably associated with strong increases in GCaMP signals. **DOI:** http://dx.doi.org/10.7554/eLife.23045.006

### Aversive learning directs the formation of excitatory responses to aversion-predicting cues

**Figure 3.**
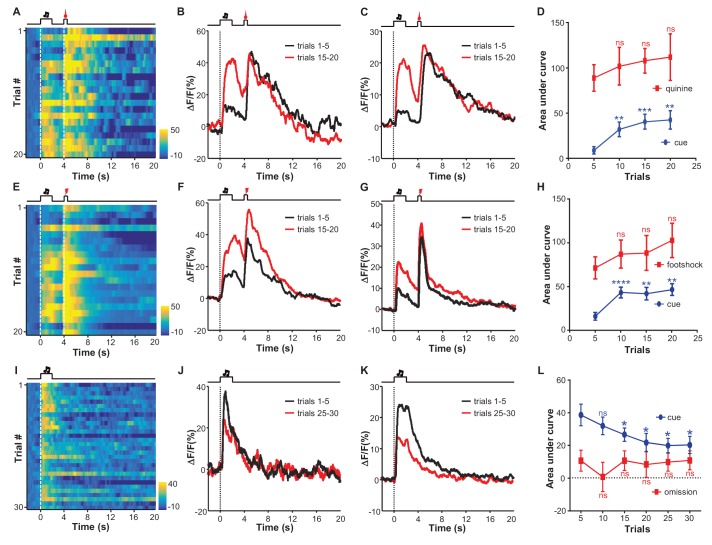
Aversive conditioning rapidly induces excitatory responses to aversion-predicting cues and omitting an unconditioned aversive stimulus slowly extinguishes previously-conditioned responses. (**A**) Heatmap representation of LHb Ca^2+^ transients within a session of cue-quinine Pavlovian conditioning. The conditioning session consisted of 20 trials. The dashed lines and timeline below indicate the timing of an auditory cue (2 s), delay (2 s), and intra-oral infusion of quinine (0.5 s). (**B**) The peri-event plot of the average Ca^2+^ transient from the same mouse shown in (**A**) during the first five trials (black) and last 5 trials of the conditioning session. (**C**) Mean Ca^2+^ transient for the entire test group (n = 9 mice). (**D**) Sum of Ca^2+^ transients for cues (0–2 s; blue line) and quinine infusion (4.0–4.5 s; red line) throughout the conditioning process. (**E–H**) LHb neurons rapidly gained responses to an auditory cue after its coupling to footshock. (**E**) Heatmap representation of Ca^2+^ transients during a conditioning session (n = 20 trials). (**F**) Mean Ca^2+^ transients across the conditioning trials for the same mouse shown in (**E**). (**G**) Mean Ca^2+^ transients for the entire test group (n = 9 mice). (**H**) Ca^2+^ responses to the auditory cue (0–2 s, blue line) increase, whereas those to the footshock (4.0–4.5 s, red line) remain largely stable during the conditioning phase (n = 9 mice). (**I–L**) The effects of omitting footshock on previously conditioned responses to the footshock-predicting cue. (**I**) Heatmap representation of Ca^2+^ transients in an extinction session (30 trials), within which we repetitively presented 30 CS cues but omitted footshock. (**J**) Mean Ca^2+^ transients for one extinction session. (**I** and **J**) correspond to the same mouse in (**E** and **F**). (**K**) Population mean of Ca^2+^ transients (n = 9 mice). Thick lines indicate the mean and shaded areas indicate the SEM. Red segments indicate statistically-significant increases from the baseline (p<0.05; multivariate permutation test). (**L**) Sum of Ca^2+^ transients during cue presentation (0–2 s; blue line) and footshock omission (4–4.5 s; red line). Each data point represents the average of 5 consecutive trials. (In D, H, L), *p<0.05; **p<0.01; ***p<0.001; ****p<0.0001; n.s., not significant; nonparametric one-way ANOVA with Geisser-Greenhouse correction for the difference between the first data point and those of the following trials. **DOI:** http://dx.doi.org/10.7554/eLife.23045.007

We sought to understand how learning might shape the responses of LHb neurons to aversive stimuli. We adopted an aversive Pavlovian conditioning paradigm, in which a 2 s auditory tone (conditioned stimulus, CS) was coupled to the delayed (2 s) delivery of a 0.5 s quinine infusion (unconditioned stimulus; US). Initially, the tone did not evoke any significant change in Ca^2+^ signals from LHb neurons (Figure 3A). With the repeated instances of tone-quinine coupling trials, the tone elicited increasingly stronger responses that peaked within 1 s and decayed following tone termination (Figure 3B). During this process, LHb neurons continued to respond to quinine infusion (Figure 3B; Figure 3C). We observed similar response patterns from all test mice (n = 9 mice). At the population level, CS-evoked responses became statistically significant between 5 and 10 trials and reached a plateau within 15 trials (Figure 3D). The strength of the quinine-evoked responses exhibited an increasing trend throughout the conditioning process, although these changes were not statistically significant (Figure 3D), suggesting that LHb neurons faithfully tracked the value of quinine-associated aversiveness during the learning process.

**Figure 3—figure supplement 1.**
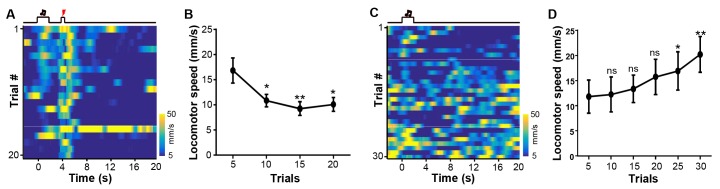
Cue-footshock associative learning changes animal locomotor behavior. (**A**) Heatmap representation of the locomotor speed of a mouse shown during a conditioning session. Same mouse as shown in Figure 3E and Figure 3I. (**B**) At the group level, animal locomotor speed changed during the footshock-predicting period (0–4 s after cue onset) across conditioning trials. Each data point represents the average of 5 consecutive trials. (**C**) Heatmap representation of the locomotor speed of the same mouse shown in (**A**) in an extinction session. (**D**) Population locomotor speed following cue presentation (0–4 s following cue onset) across extinction trials. Each data point represents the average of 5 consecutive trials. The speed was measured as the rate of body position change. (In B and D), *p<0.05; **p<0.01; n.s., not significant; nonparametric one-way ANOVA with Geisser-Greenhouse correction for the difference between the first data point and those in the following trials; n = 5 mice. **DOI:** http://dx.doi.org/10.7554/eLife.23045.008

Coupling an auditory cue to footshock influenced the responses of LHb neurons in a manner similar to that observed for quinine infusion (Figure 3E–H). Initially, the footshock (US), but not the tone (CS), elicited strong Ca^2+^ transients (Figure 3E). Within 5–10 trials, the tone preceding the footshock produced a strong increase in the Ca^2+^ signals that rose transiently and then decreased following the cue offset (Figure 3E–G). At the population level, the cue-evoked responses became significant and reached a peak within 10 trials, while the footshock-evoked responses remained largely unchanged (n = 9 mice; Figure 3H). We monitored the locomotor activity of 5 mice. In the beginning trials, mice exhibited active locomotion following the cue. Merely after 10 trials of training, their locomotor activity during the footshock-predicting period became significantly lower than the initial level (Figure 3—figure supplement 1A; Figure 3—figure supplement 1B). These behavioral changes are temporally consistent with the changes in cue-evoked activity of LHb neurons. Therefore, classical conditioning can rapidly shape the response patterns of LHb neurons, forming aversive memories after only a few trials of cue-aversion association.

We further examined whether omitting the US could extinguish the memory of the CS in the LHb. The day after initial conditioning, we applied the footshock-predicting cue but omitted footshock. Initially, the cue produced Ca^2+^ transients with amplitudes comparable to those in the conditioning session, suggesting that the aversion memory lasted for at least a day (Figure 3I). Continually omitting the footshock gradually weakened the cue-evoked Ca^2+^ signals to a significantly reduced level after approximately 20 trials, although the signals remained substantially above the baseline after 30 trials (Figure 3J–L). During the extinction sessions, animal locomotor activity became gradually increased; the change reached statistical significance after about 25 extinction trials (Figure 3—figure supplement 1C; Figure 3—figure supplement 1D). This indicates that the aversive memory of LHb neurons was subjected to reversal, but the reversal rate was slower than the conditioning rate (Figure 3D; Figure 3H; Figure 3L).

**Figure 4.**
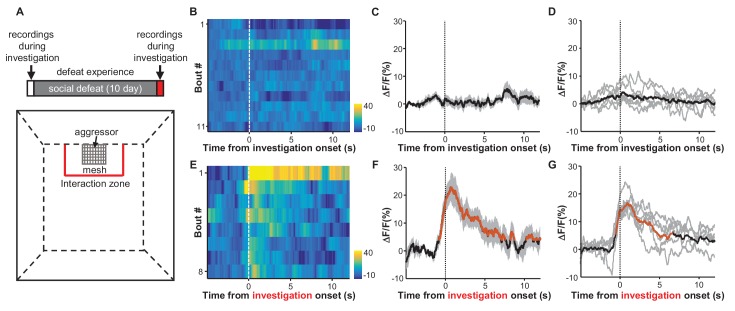
Social defeat by CD-1 aggressors induces excitatory responses of LHb neurons that were previously nonresponsive to aggressors. (**A**) Behavioral experimental paradigm. The upper panel shows the timeline of the experiments. During the first and last days (day 1 and day 12), we recorded Ca^2+^ signals of LHb neurons from a test mouse interacting with a CD-1 aggressor separated by a mesh enclosure in the social interaction arena (lower panel). During days 2-11, the test mouse was challenged with social defeat by an aggressive CD-1 mouse in the resident CD1 aggressor’s home-cage. (**B**–**D**) Heatmap representation (**B**) and averaged Ca^2+^ signals (**C**) from one test mouse and the group data (D, n = 6 mice) show that investigation of the aggressor by the test mouse did not produce any clear activation of LHb neurons. We defined an 'investigation' event as when the test mouse entered the interaction zone near the aggressor. (**E**–**G**) Following repeated social defeat over 10 days, a naive aggressor induced clear activation of LHb neurons in the test mouse. (**E**) A heatmap illustrating the response of eight consecutive investigation events of one test mouse in a behavioral session. (**F**) Average responses of the same mouse shown in (**E**). (**G**) Group data (n = 6 mice). In (**C** and **F**), thick lines indicate the mean and shaded areas indicate the SEM. In (**D** and **G**), each gray line represents data from an individual test mouse. Red segments indicate statistically-significant increases from the baseline (p<0.05; multivariate permutation test). **DOI:** http://dx.doi.org/10.7554/eLife.23045.009

We next determined how the experience of social defeat influences the responses of LHb neurons to social stimuli. We first allowed a test mouse to freely investigate a social interaction arena in which a strange CD-1 aggressor mouse was held in a mesh enclosure. We measured the Ca^2+^ signals of LHb neurons from the test mouse as it entered the defined interaction zone in proximity to the aggressor-holding enclosure (Figure 4A). Initially, LHb neurons did not exhibit any clear response to the aggressor (Figure 4B–D). Over the following 10 days, the test animal was repeatedly subjected to bouts of social defeat by a CD-1 aggressor (Figure 4A). We then re-examined the responses of LHb neurons to the aggressor within the interaction arena. Following 10 days of repeated social defeat, LHb neurons were activated significantly in the presence of an aggressor (Figure 4E–G). It is notable that these activation responses were initially strong but gradually decreased with each follow-up interaction (Figure 4—figure supplement 1), suggesting that repetitive encounters that lacked actual harm may extinguish the LHb activation that a mouse had previously associated with an aggressor.

**Figure 4—figure supplement 1.**
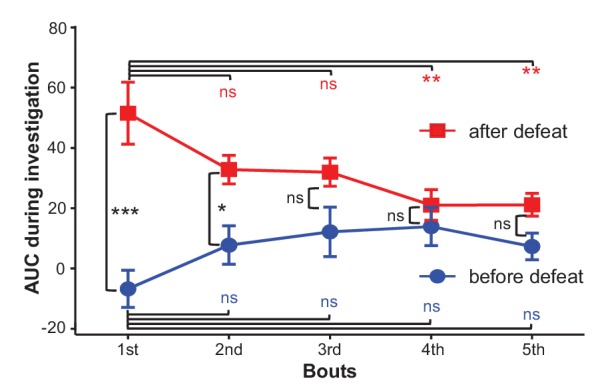
The effects of social defeat and investigation trials on investigation-associated Ca^2+^ signals of LHb neurons. During the first day, LHb neurons did not exhibit any significant response to a strange CD-1 male. After 10 daily sessions of social defeat by aggressive CD-1 males, the LHb neurons became significantly activated when the test mouse interacted with an aggressor in a mesh enclosure. ns, not significant; *p<0.05; **p<0.01; ***p<0.001; nonparametric one-way ANOVA with Geisser-Greenhouse correction for analyzing the difference across bouts and t-tests corrected for multiple comparisons using the Holm-Sidak method for analyzing the difference between before- and after- defeat (n = 6 test mice). **DOI:** http://dx.doi.org/10.7554/eLife.23045.010

### Learning shapes the inhibitory response of LHb neurons to rewards in a probability-dependent manner

**Figure 5.**
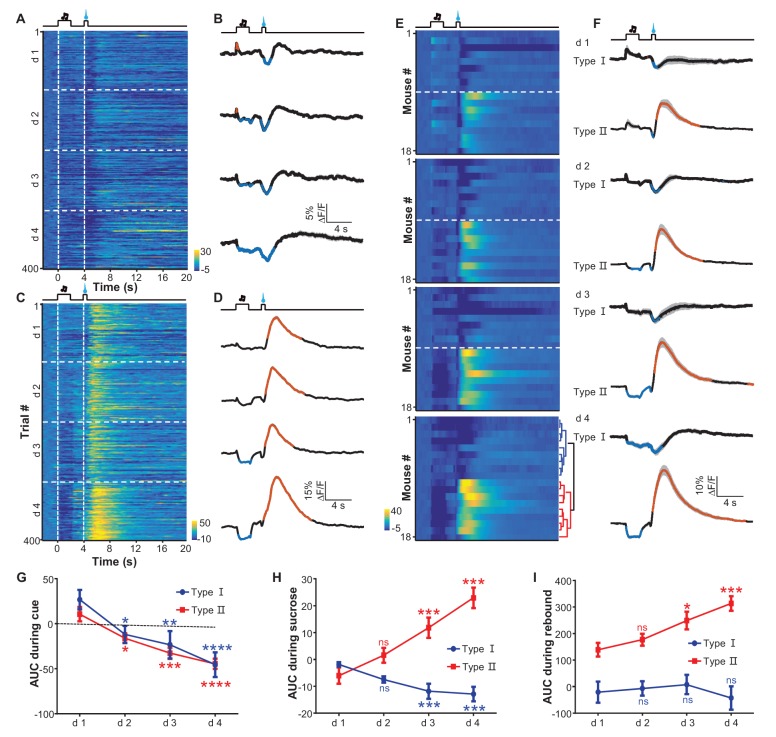
Appetitive Pavlovian conditioning changes the response patterns of LHb neurons to sucrose and sucrose-predicting cues. (**A** and **B**) An example of inhibitory Ca^2+^ responses to a cue and to a reward. The mouse was trained by coupling an auditory tone with delayed delivery of sucrose. The heatmap in (**A**) represents data from four daily sessions, each of which consisted of 100 trials. The peri-event plots (**B**) illustrate the average Ca^2+^ transients over four consecutive days (d 1–4) for the same animal shown in (**A**). (**C** and **D**) An example response pattern that included inhibition by the cue and an inhibition-then-excitation response to reward. Same conventions as in (**A** and **B**). (**E**) Heatmap illustration of the reward conditioning-associated Ca^2+^ signals from 18 individual recording sites across four conditioning sessions (days 1-4). Each row represents one recording site. We clustered the response profiles into two types, including Type I, which exhibited pure inhibitory responses, and Type II, which exhibited inhibition-then-excitation responses. (**F**) Evolution of Type I and Type II responses across the training sessions. Thick lines indicate the mean and shaded areas indicate the SEM. Red and blue segments indicate statistically-significant increases and decreases from the baseline, respectively (p<0.05; multivariate permutation test). (**G–I**) The intensity of Type I and Type II responses to the cue presentation (0–2 s; **G**), sucrose delivery (4.0–4.5 s; **H**), and post-sucrose evaluation (5–10 s; **I**) across the four training sessions. *p<0.05; **p<0.01; ***p<0.001; ****p<0.0001; n.s., not significant; nonparametric one-way ANOVA with Geisser-Greenhouse correction for the difference between day 1 and the following days. **DOI:** http://dx.doi.org/10.7554/eLife.23045.011

We used fiber-photometry methods to examine the effect of reward-Pavlovian conditioning on the activity of LHb neurons throughout the learning process. Individual mice underwent four daily training sessions, each of which presented 100 trials that coupled an auditory tone (2 s) to the delayed (2 s) delivery of a sucrose infusion (0.5 s). Initially, mouse locomotor activity increased during the cue and decreased upon sucrose delivery. The sucrose-associated decrease in locomotion became more pronounced as the conditioning continued; its timing gradually shifted closer to the cue, became statistically significant after over 100 trials, and reached a stable pattern after about 300 trials (Figure 5—figure supplement 1). The establishment of this stable conditioned behavioral response to the cue indicated successful Pavlovian conditioning. Fiber photometry of Ca^2+^ signals revealed two different response patterns from 18 mice (Figure 5—figure supplement 2A). Some recordings showed that LHb neurons did not initially respond to the auditory cue yet were inhibited by sucrose (Figure 5A; Figure 5B). In the following days, the cue gradually began to evoke a mild and sustained reduction in the strength of the Ca^2+^ signals, whereas sucrose remained effective in inhibiting LHb neurons. In an independent replication of these experiments, the recordings indicated that this conditioning similarly induced mild inhibitory responses to the cue throughout the training process (Figure 5C; Figure 5D). However, in these mice, the initial inhibition by sucrose was followed by transient increases in Ca^2+^ signals. The onset of the increase was tightly coupled to the termination of sucrose delivery. Moreover, prolonging the sucrose delivery resulted in delayed activation (Figure 5—figure supplement 2B–E). This suggests that the increase in Ca^2+^ signals represents a post-inhibitory rebound rather than feedback signals associated with reward onset.

**Figure 5—figure supplement 1.**
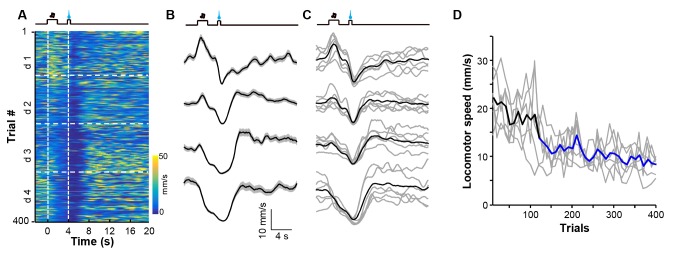
Appetitive Pavlovian conditioning changes animal locomotion during the sucrose-predicting period. (**A** and **B**) Heatmap illustration (**A**) and peri-event plots (**B**) of locomotor speed of a mouse from four consecutive conditioning sessions (d1–d4). (**C**) Mean locomotor speed for the entire test group (n = 6 mice). Black line indicates the average speed and gray lines indicate locomotor speed from each individual mouse. (**D**) Population change in locomotor speed during the reward predicting period (0–4 s after cue onset) across 400 conditioning trials. Thick line, population average; gray lines, locomotor response of each individual mouse. Each data point represents the average of 10 consecutive trials. Blue segments indicate statistically significant decreases from the baseline (p<0.05; multivariate permutation test; n = 6 mice). **DOI:** http://dx.doi.org/10.7554/eLife.23045.012

**Figure 5—figure supplement 2.**
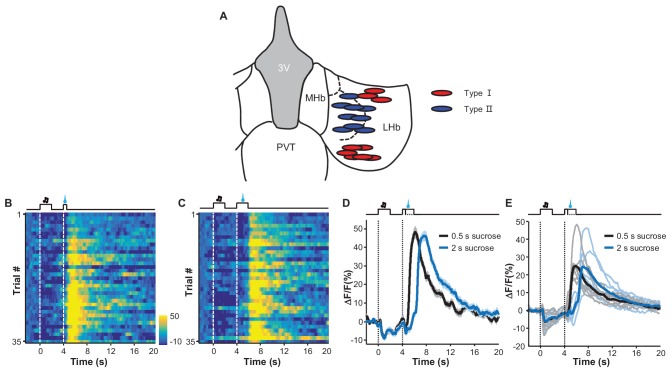
The recording sites associated with the two types of response patterns and the effect of prolonging reward delivery on the inhibition-then-excitation (Type II) response pattern. (**A**) Recording sites for Type I and Type II responses. (**B–D**) Heatmap representations (**B** and **C**) and average Ca^2+^ transients (**D**) associated with the Type II conditioned responses to sucrose delivery for 0.5 s and 2 s. (**E**) Population data (n = 6 mice). In (**C** and **D**), thick lines indicate the mean and shaded areas indicate the SEM. In (**E**), individual lines represent data from individual mice, with black and blue lines corresponding to data for, respectively, 0.5 s and 2 s of sucrose delivery duration. **DOI:** http://dx.doi.org/10.7554/eLife.23045.013

Using principal component analysis (PCA) and non-biased hierarchical clustering, we clustered the data from the recording sites of 18 mice into two major types, which we have termed Type I and Type II for simplicity (Figure 5E; Figure 5F): the Type I responses (9/18 recordings) were characterized by inhibition from both the sucrose-predicting cue and the sucrose; the Type II responses (9/18 recordings) were characterized by inhibition from the sucrose-predicting cue and an inhibition-then-excitation response to sucrose. For both types of responses, the cue itself did not evoke a significant response during the initial 100 trials of conditioning. After prolonged conditioning, the cue became increasingly effective in eliciting a response and produced a mild but statistically-significant inhibition that lasted throughout the presentation of the cue (Figure 5G). Linear regression analysis indicated that this cue-evoked inhibition became significant after about 120–150 trials, which was temporally consistent with change in locomotor activity pattern across the conditioning process. The Type I and Type II responses consistently distinct from each other; both during and immediately after sucrose infusion (Figure 5E; Figure 5F). Interestingly, the sucrose-evoked responses were also modulated by learning (Figure 5H and I). Across the four conditioning sessions, sucrose infusion produced gradually stronger inhibition in Type I responses but produced increasingly stronger excitation in Type II responses that lasted throughout the post-inhibition rebound.

Given that inactivating LHb neurons abolished the behavioral choice toward higher reward probability (Stopper and Floresco, 2014), we asked how reward probability could modulate the responses of LHb neurons. Since the inhibition-then-excitation (Type II) responses allowed us to separate the effect of the cue from a reward more easily, we focused on this response type for studying the effect of reward probability. We conditioned mice with two auditory cues (12 kHz or white noise, hereafter termed Cue1 and Cue2) that were associated with sucrose infusion, with a respective 75% or 25% chance of infusion. Before these recordings, each mouse completed six training sessions that in total presented the two cues in a pseudorandom order for 600 trials.

**Figure 6.**
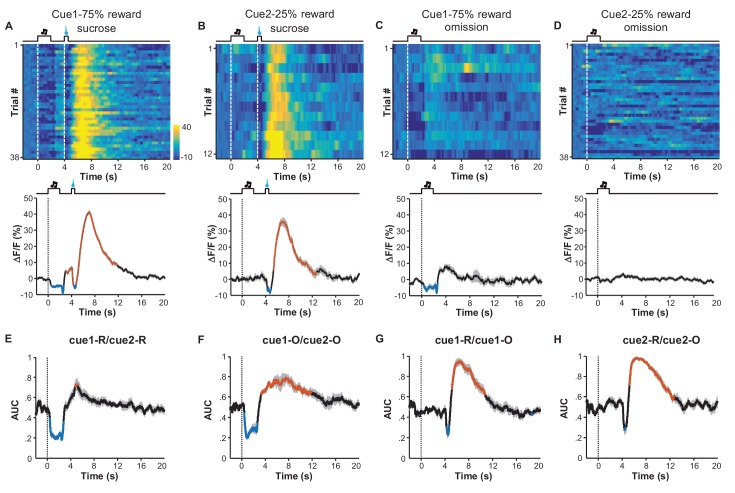
Expected probability of reward modulates the response pattern of LHb neurons. (**A–D**) Ca^2+^ signals of LHb Vglut2-expressing neurons from a mouse within the 6^th^ conditioning session, which consisted of 100 trials with either of two cues that indicated high (75%; Cue 1) or low (25%; Cue 2) probability of sucrose infusion. The heatmaps and peri-event plots of average transients illustrate the responses to reward delivery following Cue 1 (**A**), reward delivery following Cue 2 (**B**), reward omission following Cue 1 (**C**), and reward omission following Cue 2 (**D**). (**E–H**) Separable effects of reward probability and reward outcome on the Ca^2+^ signals of LHb neurons. Regardless of whether a reward was delivered (**E**) or omitted (**F**), the cue indicating higher reward probability significantly reduced Ca^2+^ signals in LHb neurons during the cue, and evoked a rebound after the cue (n = 7 mice). On the other hand, regardless of whether a reward was preceded by a cue of higher reward probability (**G**) or lower probability (**H**), actual reward delivery was associated with an initial reduction and then rebound of Ca^2+^ signals (n = 7 mice). Thick lines indicate the mean and shaded areas indicate the SEM. Red and blue segments indicate statistically-significant increases and decreases from the baseline, respectively (p<0.05; multivariate permutation test). The cue associated with 25% reward probability lacked any statistically significant effect on activity inhibition. **DOI:** http://dx.doi.org/10.7554/eLife.23045.014

We grouped the recording trials into four groups reflecting the cue type and the reward type: Cue1 with sucrose, Cue2 with sucrose, Cue1 without sucrose, and Cue2 without sucrose (Figure 6A–D). Regardless of reward outcome, the cue associated with the 75% chance of sucrose infusion produced strong inhibitory responses, whereas the cue with the 25% chance of sucrose infusion did not evoke any clear inhibition (Figure 6E; Figure 6F). Furthermore, sucrose always produced a response, regardless of the preceding cues (Figure 6G; Figure 6H). Thus, learning induces inhibitory responses to reward-predicting cues only when the cue predicts reward with a high probability.

### The reward-response profiles of individual LHb neurons

**Figure 7.**
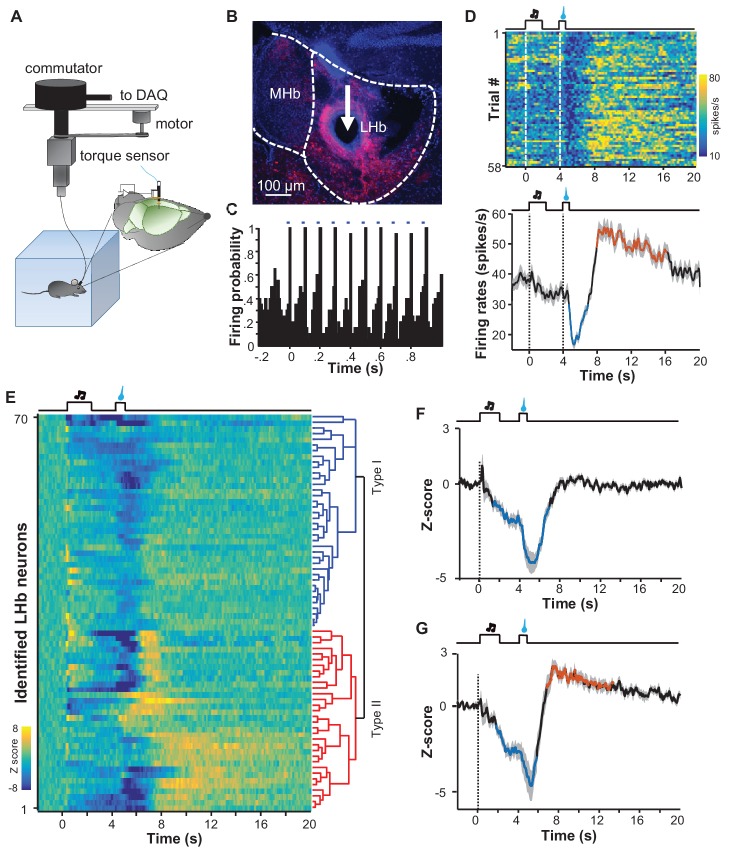
Spike firing patterns of individual LHb neurons in a reward Pavlovian conditioning task. (**A**) The schematic of setup for recording from LHb Vglut2-expressing neurons in a freely-behaving mouse. (**B** and **C**) Identification of a Vglut2-expressing neuron using optical tagging. The arrow in (**B**) points to the electrolytic lesion site targeted by an optotrode from a representative Vglut2-LHb-ChR2-mCherry mouse. Red, ChR2-mCherry. Blue, DAPI counterstaining of cell nuclei. The peri-event time histogram (PETH, bin width = 50 ms) in (**C**) shows that trains of light pulses (5 ms, 10 Hz) transiently and reliably evoked spike firing from a single unit. (**D**) Spike firing pattern of a representative LHb neuron (the same one shown in C). Upper panel, heatmap representation of the spike firing rates within the fourth daily session of Pavlovian reward conditioning. The color scale indicates the range of firing rates (spikes/s). Lower panel, PSTH of the mean firing rates (smoothed with a Gaussian kernel with σ of 50 ms). (**E**) The firing patterns of individual Vglut2 neurons (n = 70 optically-tagged cells). The standardized spike firing rates are represented as heatmaps. Each row represents the firing pattern of a single unit aligned to the cue onset. Principal component analysis indicates that the firing patterns cluster into two major subtypes. (**F** and **G**) Mean standardized firing rates of the Type I (**F**) and the Type II (**G**) response patterns of LHb neurons. Thick lines indicate the mean and shaded areas indicate the SEM. Red and blue segments indicate statistically-significant increases and decreases from the baseline, respectively (p<0.05; multivariate permutation test). **DOI:** http://dx.doi.org/10.7554/eLife.23045.015

The finding that sucrose produced a post-inhibitory increase of Ca^2+^ signals in many mice differs from previous reports that rewards predominantly suppress the activity of LHb neurons (Matsumoto and Hikosaka, 2007, 2009). To further examine the electrophysiological basis of the Ca^2+^ signals and the reward responses of individual neurons, we performed single-unit recordings from the LHb of freely moving mice (Figure 7A). Following reward conditioning, we recorded extracellular spikes with a microdrive-controlled optetrode consisting of four tetrodes and a small optical fiber (Anikeeva et al., 2011; Li et al., 2016). A motorized commutator controlled the turning of the recording cable to minimize moving-related torque (Luo et al., 2003). To confirm that we were recording LHb glutamatergic neurons, we expressed Channelrhodopsin-2 (ChR2) by infusing AAV-DIO-ChR2-mCherry constructs into the LHb of *Slc17a6-ires-Cre* mice (Figure 7B). After isolating single units, we tested whether light pulses (5 ms, 10 Hz) could reliably and rapidly elicit the firing of spikes with waveforms similar to those of spontaneous spikes (Figure 7—figure supplement 1A; Figure 7—figure supplement 1B; Figure 7C). We applied a commonly-used statistical method to analyze the significance of optogenetic tagging (Cohen et al., 2012; Kvitsiani et al., 2013; Li et al., 2016). We further made electrolytic lesion to verify the location of the recording sites in the LHb (Figure 7B).

**Figure 7—figure supplement 1.**
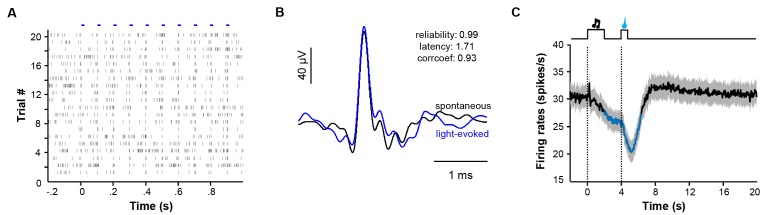
Identification of LHb neurons and the overall response pattern in a reward Pavlovian conditioning task. (**A**) Raster plot showing that trains of light pulses (5 ms, 10 Hz) transiently and reliably evoked spike firing from a single unit in a Vglut2-LHb-ChR2-mCherry mouse. Each dot indicates a spike. (**B**) Overlaying average waveforms revealed that the light-evoked spikes (blue) resembled spontaneous spikes (black). (**C**) Average PETH of the firing rates for all identified LHb neurons (n = 70 cells). **DOI:** http://dx.doi.org/10.7554/eLife.23045.016

We recorded 70 optogenetically-identified Vglut2-expressing neurons from 12 mice. Population average revealed that in general LHb neurons fired spontaneously at about 30 spikes/s, reduced their activity to about 25 spikes/s during the cue, and became further inhibited to 20 spikes/s following sucrose delivery (Figure 7—figure supplement 1C). Although there was a trend of rebound, we did not detect significant increase in firing rates for the entire group of recorded cells. However, we observed the pattern of post-inhibitory rebound in spike firing from many neurons. One such example is illustrated in Figure 7D. The firing rates of this cell stayed high at the baseline, slightly decreased following the presentation of the sucrose-predicting cue, decreased further during the delivery of sucrose, quickly rebounded after sucrose, and finally returned to the baseline in a few seconds.

Principal component analysis (PCA) and unbiased hierarchical clustering were used to classify the response patterns of the 70 optogenetically-identified LHb neurons into two major types (Figure 7E–G). Slightly more than half of neurons (37/70) exhibited inhibitory responses to both the cue and the reward, reminiscent of the aforementioned Type I responses observed via the fiber photometry of Ca^2+^ signals (Figure 7F). A substantial number of neurons (33/70) were inhibited by the cue and exhibited an inhibition-then-excitation firing pattern to sucrose, reminiscent of the Type II pattern of Ca^2+^ signals described with the fiber photometry results, above (Figure 7G). Therefore, electrophysiological recordings also revealed two response patterns that resemble the response profiles of Ca^2+^ signals.

## Discussion

Using fiber photometry and single-unit recordings, we here investigated how LHb neurons respond to aversive and reward stimuli in freely-behaving animals throughout the learning process. The similarity between the Ca^2+^ signals and the neuronal firing patterns in the reward-conditioning task supports the suitability of using fiber photometry to monitor the neuronal activity of LHb neurons. We found that (1) LHb neurons are activated by various aversive stimuli including social attack; (2) that aversive conditioning rapidly induces robust excitatory responses to aversion-predicting cues but does not affect responses to aversive stimuli; and (3) that rewards produce either pure inhibition or inhibition followed by excitation, although reward-predicting cues uniformly induce inhibitory responses in a reward probability-dependent manner.

Firstly, LHb glutamatergic neurons are rapidly and intensely activated by diverse aversive stimuli, including footshock, quinine, and social attack by an aggressor. Our observations are consistent with previous experiments showing that primate LHb neurons are excited by airpuff (Matsumoto and Hikosaka, 2009). Although airpuffs possess aversive quality, they can also briefly excite reward-encoding dopamine neurons possibly because of strong somatosensory activation (Fiorillo, 2013). It is thus important to test how LHb neurons respond to other aversive stimuli. The excitatory responses to social attacks that we observed are particularly interesting. We saw strong Ca^2+^ signals when the test mouse was attacked by an aggressor, but not when the mouse interacted with a non-aggressive social partner. Thus, social attack, rather than general social interaction, activates LHb neurons. The three aversive stimuli engage different sensory modalities and motor acts, suggesting that LHb neurons integrate various aversive inputs but do not directly link to specific motor behaviors (Baker et al., 2015). Our findings thus substantially strengthen the concept that LHb neurons encode punishment signals. LHb neurons extend particularly strong projections to midbrain GABAergic neurons that in turn inhibit reward-encoding dopamine neurons and serotonergic neurons (Varga et al., 2003; Ji and Shepard, 2007; Jhou et al., 2009; Kaufling et al., 2009; Hong et al., 2011; Lecca et al., 2011; Li et al., 2016), implying that the punishment signals from the LHb might suppress reward processing by targeting the two major modulatory centers.

Moreover, learning rapidly induces the excitatory responses of LHb neurons to aversion-predicting cues. Taking advantage of long-term recordings using fiber photometry, we examined the response profiles of LHb neurons throughout the process of aversive Pavlovian conditioning. Coupling a previously neutral auditory cue to either quinine or footshock induced the excitatory responses of LHb neurons to the cue within merely five trials, suggesting that aversion-triggered neural activity can efficiently strengthen certain synapses within the circuit from cue-responsive neurons to LHb neurons. This rapid associative learning seems likely to be particularly useful to animals for prompting avoidance behavior in response to newly perceived dangers in an ecosystem. We found that learning-induced responses to a conditioned stimulus last for at least a day, and can be substantially reversed by omitting the aversive stimuli for about 20 trials. Therefore, the activity change of LHb neurons provides a physiological correlate for stable and reversible aversive memories. The responses of LHb neurons to footshock and quinine remain stable even after the cue-induced activation reaches a peak. Therefore, LHb neurons can faithfully track the value of strongly aversive stimuli, but may not encode the difference between the predicted and currently experienced aversion (‘aversion prediction error’).

The LHb has emerged as a centrally-important brain region in the pathophysiology of depression that often involves social defeat in humans and animal models (Li et al., 2011, Li et al., 2013; Lecca et al., 2014; Proulx et al., 2014). Here, we found that the experience of social defeat induced the activation of LHb neurons to a previously-neutral aggressor. A recent study revealed that the LHb receives GABAergic inhibitory inputs from the basal forebrain neurons that are activated by aggression toward others (Golden et al., 2016). Viewed together with the study of Golden et al. (2016), our results indicate that the activation of LHb neurons may be particularly sensitive to social attack and social defeat by an aggressor, rather than by simple aggression from a test mouse toward others, suggesting that the LHb might represent a critical node in the neural circuit that mediates social defeat-triggered depression.

Previous recordings in the primate LHb have revealed only inhibitory response to rewards or reward-predicting cues (Matsumoto and Hikosaka, 2009). Both fiber photometry and single-unit recordings here demonstrated that primary rewards produced in LHb neurons a pure inhibitory (Type I) response or an inhibition followed by excitation (Type II) response. The post-inhibitory excitation might signal ‘aversiveness’ that is associated with the termination of reward stimuli, suggesting that rewards can produce distinct effects on aversion-encoding by individual LHb neurons. Regardless of the difference in reward responses of individual neurons, Pavlovian conditioning only induces inhibition to reward-predicting cues, supporting the hypothesis that LHb neurons encode negative motivational value. Moreover, conditioned responses require sufficient probability of reward, supporting a role for LHb neurons in risk aversion (Matsumoto and Hikosaka, 2009; Stopper and Floresco, 2014).

It is not clear how LHb neurons respond with an inhibition-then-excitation pattern to a reward. Given that fiber photometry methods sample the activity of a population of neurons, Ca^2+^ signals from different recording sites should be similar if individual neurons of different response profiles are evenly distributed in the LHb. The different response profiles revealed by fiber photometry thus suggest that the individual neurons exhibiting the two response patterns are likely clustered into different subregions in the LHb. The difference in activity patterns might result from distinct inputs and/or intrinsic physiological properties of neurons. One possibility lies in the expression of the Ca^2+^ channels for T-type currents, which effectively mediate post-inhibitory rebound and appear to be enriched in the medial portion of the LHb (Molineux et al., 2006; Iftinca et al., 2007). We note that with fiber photometry the response pattern of inhibition-then-excitation occurred mostly at the medial aspect of the LHb (Figure 5—figure supplement 2A), although it remains a challenge to precisely map our recording sites in freely-behaving mice to particular subnuclei in the LHb. Future genetic approaches may allow targeted recordings and manipulations to study the functional roles of such post-inhibitory excitatory responses to various rewards.

LHb neurons are often considered to be mirror-inverted versions of dopamine neurons in the midbrain ventral tegmental area (Schultz et al., 1997; Schultz, 1999; Matsumoto and Hikosaka, 2009). While our recordings clearly support the theory that the LHb responds positively to stressors and negatively to rewards, the response pattern of LHb neurons distinguishes it from a strictly inverted mirror image of dopamine neuron activity in two key ways. First, dopamine neurons encode the reward prediction error such that, after reward conditioning, they respond strongly to reward-predicting cues but do not respond to the reward itself. In contrast, LHb neurons maintain their responses to the unconditioned stimuli even after they develop strong responses to the aversion-predicting cue. Second, after learning, dopamine neurons become phasically excited by conditioned stimuli (Schultz et al., 1997; Eshel et al., 2015), whereas LHb neurons continue to be inhibited by reward-predicting cues throughout the cue and during the delay period before reward delivery. Thus, through GABAergic relays in the midbrain, LHb signals are integrated with other inputs to the VTA to generate a code of reward prediction error that differs from a simple inversion of LHb response pattern.

Collectively, our recordings from freely-behaving mice demonstrate that LHb neurons are activated by diverse stressors and respond to rewards with two distinct patterns. Moreover, associative learning can condition LHb responses to cues that predict aversion or reward in a bidirectional manner. Given that depressive behaviors involve abnormal responses to stressors and the hyperactivity of the LHb (Hikosaka, 2010; Sartorius et al., 2010; Lecca et al., 2014), our results support the idea that suppressing LHb activity may represent a potentially effective approach for treating depression.

## Materials and methods

### Mice

Animal care and use followed the institutional guidelines of the National Institute of Biological Sciences (NIBS), Beijing (Approval ID: NIBSLuoM15C) and the Regulations for the Administration of Affairs Concerning Experimental Animals of China. *Slc17a6-ires-Cre* mice (Jackson Laboratory *Slc17a6*<tm2(cre)Lowl>/J) were bred and maintained at the specific-pathogen-free mouse facility of NIBS with controlled temperature (22–25°C) and a 12/12 hr photoperiod with *ad libitum* water and standard mouse chow. All experiments were performed on adult mice (8–16 weeks of age) of either sex. After surgery, mice were housed with a reverse photoperiod (light off at 8AM) for at least one week prior to further experiments.

### Surgery and virus injection

AAV vectors carrying DIO-EmGFP or DIO-GCaMP6m constructs were packaged into the AAV2/9 serotype with titers of 1–5 × 10^12^ viral particles/ml. These plasmids were constructed by replacing the coding region of ChR2-mCherry of the pAAV-EF1a-DIO-hChR2(H134R)-mCherry plasmid (Addgene plasmid # 20297, a gift from Dr. K. Deisseroth) with a sequence encoding an enhanced form of membrane GFP (Addgene Plasmid #14757, a gift from Dr. C. Cepko) or GCaMP6m (Addgene Plasmid #40754, a gift from Dr. D. Kim).

Mice were anesthetized with pentobarbital (i.p. 80 mg/kg) and then mounted in a stereotaxic holder and were kept warm with a heating pad. A piece of scalp was cut off to expose the skull. After thoroughly cleaning the skull with 0.3% hydrogen peroxide solution, a small craniotomy (coordinate AP/DV/ML: −1.6/–2.7/−0.5 mm) was made through the skull for virus injection. Using a microsyringe pump (Nanoliter 2000 Injector, WPI), AAV vector (300 nL) was injected slowly (40 nL/min) into the LHb via a glass pipette. The glass pipette was left in place for five minutes after injection and then slowly withdrawn. An intra-oral cheek fistula was implanted in mice following a previously-described procedure (Li et al., 2016). Briefly, a small incision was made in the cheek (lateral and rostral to the first molar) and another incision was made in the scalp. A piece of soft Silastic tubing (30 mm in length, 0.30 mm I.D., and 0.46 mm O.D.; Dow Corning) was subcutaneously inserted to a depth of 2 mm into the oral cavity through the incision site. An L-shaped 26-gauge (O.D. 0.48 mm) stainless steel tub was connected to the Silastic tubing and was imbedded beside the ceramic ferrule (see below). A piece of polyethylene tubing (10 mm in length, 0.4 mm I.D., 1.1 mm O.D.) was fitted to the exposed end of the L-shaped tubing. A steel plug was inserted to the exposed end of the polyethylene tubing to prevent blockage.

### Fiber photometry

Following AAV injection, an optical fiber (230 µm O.D., 0.37 NA; Shanghai Fiblaser) was placed in a ceramic ferrule (2.5 mm O.D., 126 µm I.D.) and inserted toward the LHb. The ceramic ferrule was affixed with a skull-penetrating M1 screw and with dental acrylic. To enable recovery and AAV expression, mice were housed individually for at least 10 days following virus injection.

To record fluorescence signals, a beam from a 488 nm laser (OBIS 488LS; Coherent) was reflected with a dichroic mirror, focused with a 10× objective lens (NA = 0.3; Olympus), and then coupled to an optical commutator (Doric Lenses). An optical fiber (230 μm O.D., NA = 0.37; 2 m long) guided the light between the commutator and the implanted optical fiber. To minimize GCaMP bleaching, the laser power was adjusted at the tip of optical fiber to a low level (0.03–0.04 mW). The GCaMP fluorescence was filtered with a GFP bandpass filter and collected with a photomultiplier tube (R3896; Hamamatsu). An amplifier converted the PMT current output to a voltage signal, which was further filtered through a low-pass filter (40 Hz cut-off; Brownlee 440). The analog voltage signals were digitalized at 500 Hz (Power 1401 digitizer, CED) and sampled with Spike2 software (CED).

### In vivo electrophysiological recording and optical tagging

The protocols for the single-unit recording and optical tagging techniques have been detailed elsewhere (Li et al., 2016). Briefly, the optetrode used for recording was comprised of four tetrodes (impedance 250–500 KΩ) and one optical ferrule (125 µm diameter, NA = 0.37). The optetrode was inserted through a steel tube (10 mm in length) with the tetrode tips extending 500 µm away from the optical fiber. After injecting the virus, we gradually lowered the optetrode to a depth of 0.5 mm above the LHb. A silver wire (127 μm dia) was attached to three skull-penetrating M1 screws with silver paste, serving as ground. The microdrive was secured to the skull with dental acrylic.

Extracellular spiking signals were amplified (1000×) with a 16-channel amplifier with a built-in bandpass filter (0.5–3.6 kHz). For each recording session, a channel that did not exhibit salient spike signals was selected for use as a virtual ground to minimize movement artifacts. Analog signals were digitized at 25 kHz (Power1401 digitizer) and sampled with Spike2 software. A 25-channel commutator (Crist Instruments) was rotated using a torque-controlled servomotor to minimize torque of the recording cable (Luo et al., 2003). At the end of each recording session, the optetrode was lowered 60 µm by manually turning an M1 screw in the microdrive. Once the optetrode was judged to be outside the LHb, we stopped recording and moved the optetrode to the predetermined LHb location. An electrolytic lesion site was introduced via DC current injection through two of the tetrodes (15–20 s, 100 μA). The animals were then deeply anesthetized with an overdose of pentobarbital. After fixation with 4% formaldehyde, the mouse brains were cut into 50 μm coronal sections and examined to verify the recording sites.

Optical tagging was used to assess the cell type of the recorded single units. Low intensity laser pulses (5 ms, 10 Hz) were delivered to evoke spike firing from ChR2-expressing neurons. We calculated the correlation coefficient of spike waveforms for spontaneous spikes and evoked spikes (*C*), and chose cells with C values > 0.85. To ascertain whether or not light stimulation directly evoked spike firing, we determined the latency of evoked spikes after light onset (*L*) and evaluated the reliability of light-evoked spiking within 10 ms from light onset (*R*). We then determined the *p* value by comparing the distribution latencies of light-evoked spikes and a bootstrapped distribution of latencies of spontaneous spikes (Kvitsiani et al., 2013). Units with p<0.001 were considered to be optically-tagged neurons (Cohen et al., 2012; Kvitsiani et al., 2013).

### Behavioral tasks

#### Intra-oral infusion of quinine and sucrose and Pavlovian conditioning

A peristaltic pump (AniLab) was used to infuse 10 μL of either quinine (5 mM) or sucrose (5% w/v) through Silastic tubing into the oral cavity (speed 20 μL/s). The inter-trial interval durations were randomly set in a range between 110 and 130 s for quinine and in a range between 20 and 40 s for sucrose. For Pavlovian conditioning, an auditory tone (4 kHz for quinine and 12 kHz for sucrose, sine wave, 70 dB, 2 s) was presented for 2 s followed by 2 s delay and then 0.5 s (i.e., 10 µL) of quinine or sucrose infusion. Each daily training session consisted of 20 cue-quinine trials or 100 cue-sucrose trials, and each mouse underwent four training sessions each day. The locomotion of the test mouse was simultaneously videotaped with an overhead infrared camera during each recording session. The timing for stimulus delivery was controlled through an IC board (Arduino Uno R3) using an in-house-developed MATLAB program. Note that water was withheld from the post-surgery mice that underwent treatments involving sucrose.

To examine the effect of reward probabilities, we trained mice with two auditory cues (12 kHz or white noise) that were associated with sucrose infusion, with a respective 75% or 25% chance of infusion. Within each daily session, these two auditory cues were individually presented 50 times in pseudorandom order; each animal underwent six training sessions.

#### Footshock and cued fear conditioning

A mouse was placed in an acrylic box (25 × 25 × 30, L × W × H in cm) with a metal grid floor that delivered footshock currents (0.6 mA scrambled, 0.5 s). The conditioning session consisted of 30 trials that coupled an auditory conditioned stimulus (CS; 8 kHz, sine wave, 70 dB, 2 s) to the delayed presentation (2 s) of an unconditioned stimulus (US; 0.5 s footshock; random inter-trial intervals 20–40 s). In the extinction session of the following day, we presented 30 CS cues but omitted the footshock. We monitored the locomotor behavior of the test mice with an overhead infrared camera under both conditioning and extinction session.

#### Social interaction

The test mice were housed individually for at least one week after surgery. In the male-female interaction sessions, we introduced a female with sexual experience into the home cage of the test male mouse. A recording session lasted 30–60 min and the behavior of the test mouse was videotaped with an overhead infrared camera. Interaction onset was characterized as the chemoinvestigation or mounting that lasted for at least 3 s. In the male-male fighting sessions, we introduced a test male mouse into the home cage of an aggressive CD-1 mouse. Fighting onset was defined as the moment when an aggressive mouse attacked the test mouse for a period lasting at least 3 s.

#### Social defeat

The social defeat paradigm used here has been detailed previously (Golden et al., 2011). The test mouse was exposed to social defeat stress for 10 min in the resident CD1 aggressor’s home-cage on 10 consecutive days. After 10 min of social defeat, the test mouse was transferred across the perforated divider to the opposite compartment and housed with the resident CD1 aggressor for the remainder of the 24 hr period. Test mice were rotated among different CD1 aggressors across defeat days so that a test mouse would not habituate to a single CD1 aggressor. On the day 1 and day 12, we videotaped and recorded calcium signals from the test mouse when it interacted with a novel CD-1 aggressor separated by a mesh enclosure in the social interaction arena (42 × 42 × 30, L × D × H in cm).

### Histology and immunostaining

Mice were deeply anesthetized with an overdose of pentobarbital and then transcardially perfused with 0.9% saline followed by 4% paraformaldehyde in phosphate-buffered saline (PBS). After post-fixation overnight, the mouse brain was cryoprotected with 30% sucrose for two days, and then the brain was sectioned coronally (40 µm thick) with a cryostat (Leica CM1900). For immunofluorescent staining, the sections were blocked with 3% BSA in PBS with 0.3% Triton X-100 and subsequently incubated with a rabbit polyclonal antibody to GFP (1:400; Abcam; RRID:AB_303395) at 4°C overnight. After washing with PBS, the sections were incubated with Cy2-conjugated goat anti-rabbit IgG (1:500; Jackson ImmunoResearch; RRID:AB_2338021) for 2 hr at room temperature. Finally, sections were cover-slipped with 50% glycerol mounting medium. We conducted postmortem analysis to verify the expression of GCaMP6m and to evaluate the placement of the optical fiber. We only analyzed data from mice with the tip of the optical fiber clearly located in the LHb.

### Data analysis and statistical tests

Fiber-photometry recording data were exported as MATLAB. Mat files from Spike2 software for further analysis. All the raw data were smoothed with a moving average filter (20 ms span) and then segmented and aligned according to the onset of behavioral events within individual trials or bouts. The fluorescence change (*ΔF/F*) values were calculated as *(F−F_0_)/F_0_*, where *F_0_* is the baseline fluorescence signals averaged over a 1.5 s-long control time window (typically set 0.5 s) prior to a trigger event. To analyze the responses during social interaction, the control time window was set 3.5 s before interaction onset to minimize potential chasing-induced effects. *ΔF/F* values are presented as heatmaps or as average plots with a shaded area indicating the SEM. Mouse locomotor activity was analyzed using a custom video tracking software developed in house using MATLAB. We plotted the locomotor speed during the 4 s time window following cue onset (0 – 4 s) across individual trials in a behavior session. We then averaged the value per five trials for aversive conditioning and per ten trials for appetitive conditioning.

For the in vivo electrophysiological data, the spikes were sorted off-line with the Spike2 program. Single units were isolated using principal component analysis (PCA) of the spike waveforms that had signal-to-noise ratios of at least 2:1. PETH of spike firing rates (bin width 50 ms) were smoothed with a Gaussian kernel (σ = 50 ms) and then presented either as heatmaps or as average plots. To calculate standard scores, we used the mean firing frequency of a control period. Hierarchical clustering was carried out by reducing the dimensionality of standardized firing activity via principal component analysis (PCA). The first three major principle components (PCs) were then used to calculate a Euclidean distance metric. The complete agglomeration method was applied to build the hierarchy of clusters. Minor adjustments were made by sorting the clusters in the descending order based on the total *Z*-score values between 5 and 10 s from cue onset.

We applied multivariate permutation tests to analyze the statistical significance of the event-related fluorescence (ERF) change or peri-event time histograms (PETH) of spike firing rates (1000 permutations, α level of 0.05). The null distribution was retrieved from the maximum absolute *T*-score of all permutations to correct multiple comparisons in two-tailed tests. A series of inferential p values at each time point were generated and the results were superimposed on the average ERF or PETH curves with red and blue lines indicating statistically significant (p<0.05) increases or decreases, respectively.

We plotted receiver operating characteristic (ROC) curves and calculated the area under the curve (AUC) for ERF throughout each trial by comparing the ERF of a 200 ms test window (50 ms advance step) to those in a control time window (200 ms) that occurred 1.8 s preceding the trial onset (−2 to −1.8 s). ROC values >0.5 indicate activation, and values <0.5 indicate inhibition. Differences in the ROC values between the Cue1 and Cue2 in Figure 6E–H were calculated by comparing ERF numbers during the same time windows (200 ms width, 50 ms advance step) throughout the entire trial. ROC values of 1 indicate complete selectivity for the Cue1 stimulus, and ROC values of 0 indicate complete selectivity for the Cue2 stimulus. Permutation tests with 1000 permutations were used to determine the statistical significance of the response strength and selectivity of aversive stimuli or rewards. We performed Kolmogorov–Smirnov tests for the statistical significance of the differences between the cumulative probability distributions. Similarly, we calculated the AUC for event-related fluorescent changes (*ΔF/F*) as the sum of Ca^2+^ transients.

We performed hierarchical clustering of the reward-related responses in three steps. We first applied principle component analysis (PCA) to reduce the dimensionality of standardized GCaMP signals and firing activity. We then used the first three major principle components (PCs) to define a Euclidean distance metric. Finally, we applied the complete agglomeration method to construct the hierarchy of clusters and plot dendrograms in MATLAB.
