# Supplementary material for: Learning shapes the aversion and reward responses of lateral habenula neurons
Source: eLife. 2017 May 31;6:e23045. doi: 10.7554/eLife.23045 (PMC5469615; doi:10.7554/eLife.23045)
Supplement: Related texts

# Article commentary: Decision letter

**DOI:** 10.7554/eLife.23045.019

**Affiliations:** Columbia University in the City of New York, United States

In the interests of transparency, eLife includes the editorial decision letter and accompanying author responses. A lightly edited version of the letter sent to the authors after peer review is shown, indicating the most substantive concerns; minor comments are not usually included.

Thank you for submitting your article "Learning Shapes the Aversion and Reward Responses of Lateral Habenula Neurons" for consideration by *eLife*. Your article has been reviewed by three peer reviewers, one of whom is a member of our Board of Reviewing Editors and the evaluation has been overseen by a Senior Editor. The reviewers have opted to remain anonymous.

The reviewers have discussed the reviews with one another and the Reviewing Editor has drafted this decision to help you prepare a revised submission.

The reviewers found the work novel, especially the learning dynamics, but raised several issues (summarized below) that should be addressed in a revised version of the study.

1) Regarding the point about ecologically relevant stimuli, the reviewers don't think that foot shocks and quinine delivered through a surgically implanted cannula are any more ecologically relevant than air puffs. Therefore these statements need to be toned down, and perhaps use the findings to state instead that the results demonstrate generality in the response LHb neurons to aversive events. The emphasis should be more on the learning, and the dynamics.

2) – If the social defeat dataset is to be included, then the authors need to redo these experiments to clearly demonstrate that the signals come from defeat, and with more appropriate controls (not a female mouse).

3) – The fact that this is a genetically defined population of LHb neurons should be emphasised: it is a strength but also a specificity.

4) – There are a few analyses (at least), or maybe a few quick experiments that the authors would need to do to characterise better the dynamic responses to appetitive stimuli and appetitive predicting stimuli during learning. It seems that characterisation of the positive responses, and potential source of the positive responses would need to be further dissected or at least discussed.

Reviewer's comments:

Reviewer #1:

This study presents in my view two new findings:

1) It shows that LHb neurons respond to "innately" aversive stimuli, like quinine or footshock, and to complex ecologically relevant stimuli like social defeat.

2) – It shows complexity in how these neurons respond to CSs predicting aversive stimuli, and also to appetitive stimuli, that challenge a bit the view that these neurons encode a reward prediction model symmetric to the dopamine neurons.

This second point requires a bit further characterization, or at least discussion.a) It is important to understand if in the Pavlovian conditioning to aversive cues the authors model is that further training should lead to a full response of LHb neurons to the CS and disappearance of the response to the US, or if the authors think LHb neurons will always respond to "innately aversive stimuli". b) The complex and dynamic responses to appetitive stimuli are interesting. Do the authors think these are really different cell populations? or is it that after receiving an appetitive stimulus some neurons encode the end of reward as mildly aversive?

Also, in panel E of Figure 7, one can appreciate many neurons with just a positive response to cue onset. Can the authors cluster the response just to cue onset and just to US delivery in that figure?

Reviewer #2:

In this manuscript, Wang et al. present through a combination of multiple behaviors and in vivo neuronal activity recording techniques an insight on how LHb bidirectionally encodes emotional status. Specifically, they applied fiber photometry, single unit recording and opto tagging to track the response pattern of LHb neurons in freely behaving mice. They found that bitterness, pain and social defeat increase neuronal activity while reward evokes either a pure inhibition or a transient inhibition followed by an excitation in LHb glutamatergic neurons.

The paper is of great general interest as it not only substantially strengthened the concept that LHb neurons encode aversive signals, but also proved that they integrate various aversive inputs. Moreover, by using combination of fiber photometry and optrode in vivo single unit recording, they reliably demonstrated that reward evoked two patterns of neuronal activity, which uncovers a new response pattern of LHb neurons to primary rewards. Hence, I would like to recommend its publication in *eLife*. However a few points need to be addressed:

1) The excitatory response in Figure 6A onsets right after the termination of the reward CS and US (~0.5s delay). It seems to occur faster than the excitatory spike response in Figure 7 (~2 sec delay). Can the authors comment why the Ca^2+^ response already becomes positive when the spikes are still inhibited?

2) For the cause of the excitatory signals following inhibition in response to reward, another potential source could be inputs from other reward-responding brain areas (e.g. DRN, VTA). Can the authors extend the period of reward delivery to see how that affects the onset of excitatory responses? If it's from the feedback, the onset of the excitatory response will perhaps not be delayed in relation to the reward onset. If it's the rebound, the onset will be delayed in relation to reward termination correspondingly.

3) The authors performed the "social interaction" with a female mouse as a control of social defeat experiment. However, females are often considered as a social reward. As shown in Figure 2H, there are also bidirectional responses of LHb neurons from the onset of introduction of female mice. I would suggest, first, use a non-aggressive male mouse (e.g. cagemate of the test mouse) for the 'social interaction' control; and second, check whether responses to the female can also be grouped into two types, as for the responses toward sucrose.

4) In paragraph two, subsection “The reward profiles of individual LHb neurons”, the authors showed that the average of LHb spiking rate is about 30Hz, which is much higher than previous reported (5~10 Hz, Aizawa, 2014; Golden et al., 2016). Please explain the discrepancy. What percentage of the recorded LHb neurons are glutamatergic based on optotagging?

Reviewer #3:

The manuscript by Wang and colleagues investigates the dynamics of activity in lateral habenula neurons (LHb) in freely moving mice, during the presentation of aversive sensory cues, and Pavlovian conditioning with aversive and rewarding unconditioned stimuli. They mainly use fiber photometry from LHb Vglut2 neurons expressing GCamp6, and show an increase in activity upon aversive stimulation and expectation of aversion, and a decrease in activity upon expectation of reward. They further show that conditioning of neural responses happens over a small number of trials, and that conditioning does not change the response to unconditioned stimuli. Most of the data in the manuscript support the main claims, but I have some concerns about novelty and about the results on social defeat. The main justification for the work, as detailed in the Introduction, is that while previous studies in primates have measured neuronal activity in LHb neurons in response to aversive air puffs during Pavlovian conditioning (e.g.: Matsumoto and Hikosaka 2009), the current study addresses "ecologically important stressors". I'm afraid that I don't think that foot shocks and quinine delivered through a surgically implanted cannula are any more ecologically relevant than air puffs. They are aversive stimuli, and as such generate largely the same activity profile in LHb as it has been previously reported in primates. Indeed, the majority of the findings are very similar to the results reported by Matsumoto 2009, though here performed here in freely mice. The main advances in my opinion, apart from the replication of the primate results in a genetically tractable model species, and in a molecularly-defined population of cells, which is certainly useful for the field, are the signal dynamics during learning, and the observation that conditioning happens over a small number of trials while not changing the response to the unconditioned stimulus. In addition, the social defeat paradigm is potentially interesting, and much more "ecologically relevant", but I don't think that the experimental design is appropriate to support the claims. In the current form, the authors cannot distinguish fighting-related signals from social defeat. As the only signals analyzed are during the fighting bout, it could well be that LHb neurons are signaling fighting as an aversive event, but the signal is unrelated to the outcome of the fight. A basic control would be to analyze signals in fighting bouts that the resident mouse won, but better yet to separate fighting from the social interaction with the aggressor. This could be done by having for example an initial period of social interaction where the two mice are separated by a mesh to prevent fighting, followed by the fighting trial. If LHb neurons respond to social defeat, then the prediction is that there should be an activity increase during interaction with an aggressor that has defeated the subject repeatedly. Another main comment is that it is not clear at any point in the paper how the behavior correlates with the signals. For example, for the trials shown in Figure 3E, was there freezing in response to the conditioned stimulus when there was LHb signal; or in Figure 3I, how did freezing track the reduction of LHb signals? Overall I think that if the major issues are addressed and the report of focuses on the main advances, this is a potentially interesting paper that should stimulate further studies in the field.

[Editors' note: further revisions were requested prior to acceptance, as described below.]

There is one important outstanding issue, about whether or not behaviour was assessed during the experiments, especially during the learning (Pavlovian conditioning) experiments. It does appear that although the behaviour was conducted in the dark, some behavioural measurements were taken. However, it is unclear if this was done for all the animals and if movement can be assessed or not.

It would very important to clarify this point before we proceed further.

Reviewer #2:

The authors have satisfactorily addressed all my comments.

Reviewer #3:

The authors have taken my comments on board and made a serious effort to address them. The manuscript has benefited from editing, and the toning down of the ecological relevance of the stimuli used provides a more realistic account of the study. The new experiments on the social defeat paradigm are great. These are now appropriately controlled for, and the results on the exposure to the aggressor before and after social defeat make a clear case for an aversion signal (instead of a fighting-related signal) – the data are convincing, and the dynamics observed interesting.

I have, however, one major comment remaining. In response to my request for correlating neuronal activity with behavior, the authors reveal that they have not monitored behavior for most experiments. I had not realised this, and I am slightly perplexed: learning and Pavlovian conditioning are behavioral constructs, what evidence do the authors have that mice actually learned anything? While there is little doubt that a footshock or quinine are aversive, the question is whether the conditioning paradigms used do lead conditioning. If the authors cannot provide convincing evidence that this was the case, then the consequence in my view is that the terms learning and Pavlovian conditioning have to be removed altogether from the manuscript. The authors can state that they have used associative conditioning protocols, but they cannot refer to this as learning, and must make clear that the changes are only detectable at the neural activity level, but not at the behavioural level. The latter outcome is still potentially interesting, but it does require the ability to quantify the presence or absence or behavioral conditioning. If this is not possible, I have to say that my enthusiasm for the paper is severely mitigated (on a similar line, what exactly is the "reward probabilistic discriminative task"? One thing is to expose mice to rewards of different probability, another is to require them to perform a task contingent on their ability to estimate these probabilities. I have the impression that the authors did the former, and if so, this is not a "task").

# Reply: Author response

**DOI:** 10.7554/eLife.23045.020

Reviewer #1:

This study presents in my view two new findings:

1) It shows that LHb neurons respond to "innately" aversive stimuli, like quinine or footshock, and to complex ecologically relevant stimuli like social defeat.

2) It shows complexity in how these neurons respond to CSs predicting aversive stimuli, and also to appetitive stimuli, that challenge a bit the view that these neurons encode a reward prediction model symmetric to the dopamine neurons.

This second point requires a bit further characterization, or at least discussion.a) It is important to understand if in the Pavlovian conditioning to aversive cues the authors model is that further training should lead to a full response of LHb neurons to the CS and disappearance of the response to the US, or if the authors think LHb neurons will always respond to "innately aversive stimuli".

Figure 3A-H show the data on aversive Pavlovian conditioning. We repetitively applied 20 trials of cue-quinine or cue-footshock pairs. The responses to CS (cue) quickly appeared and became largely stabilized, whereas the response to US (quinine or footshock) even showed a trend of further increase. Theoretically, we could test whether the US response will persist following the application of the CS-US pairs for many more trials, although the consideration of animal welfare and the institutional guidelines require us to reduce the number of aversive trials to as low a number as possible. Based on the current experimental results, we believe that LHb neurons continue to respond to innately aversive stimuli (US) even after the establishment of excitatory responses to the preceding cues (CS).

*b) The complex and dynamic responses to appetitive stimuli are interesting. Do the authors think these are really different cell populations? or is it that after receiving an appetitive stimulus some neurons encode the end of reward as mildly aversive?*

Reviewer 1 here raises a very intriguing question. First, we noted that the recoding sites of Type I and Type II response patterns are separated with rough topography. Second, we found that the response profiles of single units can be clustered into two patterns that resembled the Ca^2+^ signals measured with fiber photometry. These observations lead us to propose that there exist two cell populations that underlie, respectively, the Type I (pure inhibition) and Type II (inhibition-then-excitation) responses. This interpretation is compatible with reviewer 1’s proposal that the rebound might signal “aversiveness” associated with the termination of a reward (Discussion section paragraph four).

Also, in panel E of Figure 7E, one can appreciate many neurons with just a positive response to cue onset. Can the authors cluster the response just to cue onset and just to US delivery in that figure?

Reviewer 1 insightfully noted that many LHb neurons were briefly activated following cue onset (Figure 7). Fiber photometry also revealed brief and small Ca^2+^ transients immediately following the cue, especially during the initial phase of conditioning (Figure 5A). In Figure 7, we used PCA to cluster the response of single neurons in the form of a dendrogram. Following reviewer 1’s suggestion, we have clustered the responses simply to cue onset and simply to US delivery (Author response image 1). Because we did not observe any clear pattern with the new clustering criteria, we chose not to show these re-arranged heatmaps in the revised formal figures.

**Author response image 1.** Heatmaps represent the firing patterns of individual Vglut2 neurons clustered by response intensities during cue onset (**A**) and during US delivery onset (**B**). n = 70 optically-tagged cells. **DOI:** http://dx.doi.org/10.7554/eLife.23045.017

Reviewer #2:

[…]

1) The excitatory response in Figure 6A onsets right after the termination of the reward CS and US (~0.5s delay). It seems to occur faster than the excitatory spike response in Figure 7 (~2 sec delay). Can the authors comment why the Ca^2+^ response already becomes positive when the spikes are still inhibited?

This is indeed quite intriguing. As reviewer 2 correctly pointed out, for the inhibition-then excitation response pattern, the rise of Ca^2+^ signals often occurred earlier than the increase in spike firing rates. A parsimonious interpretation lies in the notion that the process underlying the increase of Ca^2+^ signals, such as the activation of a Gq-coupled GPCR and/or the opening of Ca^2+^-permeable channels, is somehow combined with an inhibitory process, such as the ongoing inhibitory postsynaptic currents. Spike firing rates will evidently increase only when the former overcomes the latter. We agree that in future it will be highly interesting to dissect the exact molecular and cellular mechanisms underlying the difference in the delay between Ca^2+^ signals and spike firing.

2) For the cause of the excitatory signals following inhibition in response to reward, another potential source could be inputs from other reward-responding brain areas (e.g. DRN, VTA). Can the authors extend the period of reward delivery to see how that affects the onset of excitatory responses? If it's from the feedback, the onset of the excitatory response will perhaps not be delayed in relation to the reward onset. If it's the rebound, the onset will be delayed in relation to reward termination correspondingly.

This is a very insightful suggestion. In the course of our revision process, we examined the effect of a short reward (0.5 s, 5% w/v, 5 μl sucrose) and a long reward (2 s, 5% w/v, 20 μl sucrose). These two reward sizes were randomly delivered. Regardless of the duration of sucrose delivery, the onset of the excitatory response was tightly coupled to the end of reward delivery (Figure 8—figure supplement 1B-1E). This observation indicates that the increase of Ca^2+^ signals arises from a process associated with reward termination, rather than from feedback signals associated with the reward onset.

3) The authors performed the "social interaction" with a female mouse as a control of social defeat experiment. However, females are often considered as a social reward. As shown in Figure 2H, there are also bidirectional responses of LHb neurons from the onset of introduction of female mice. I would suggest, first, use a non-aggressive male mouse (e.g. cagemate of the test mouse) for the 'social interaction' control; and second, check whether responses to the female can also be grouped into two types, as for the responses toward sucrose.

We agree. In our revision process, we used cage-mate males of the defeat test mouse for the social interaction control. Similarly, no clear Ca^2+^ signals were detected from test mice when they interacted with the cage-mate mouse. We have preferred to exclude the female-male interaction data from the revised study, as we agree that this control was inappropriate.

4) In paragraph two, subsection “The reward profiles of individual LHb neurons”, the authors showed that the average of LHb spiking rate is about 30Hz, which is much higher than previous reported (5~10 Hz, Aizawa, 2014; Golden et al., 2016). Please explain the discrepancy. What percentage of the recorded LHb neurons are glutamatergic based on optotagging?

We thank reviewer 2 for highlighting the discrepancy between our results and some of the previous reports. Single-unit recordings from the LHb of freely-moving mice have been reported only rarely to date. Our observations are consistent with previous recordings in behaving primates (Matsumoto and Hikosaka, 2007 & 2009), which report that the average of LHb spike firing rate is quite high (about 30Hz). Golden’s work demonstrated that the firing rate of LHb neurons was quite low (2Hz) from both slice recordings and in vivo recordings in anaesthetized mice. Using the similar recording techniques, Aizawa’s study showed that the firing rate was almost 10 Hz. So, a possible explanation of this discrepancy may be the different recording techniques. When optotagging, we performed biased recordings only when light-evoked spikes were detected in one channel. The percentage of successful optotagging was approximately 40%.

Reviewer #3:

[…] I'm afraid that I don't think that foot shocks and quinine delivered through a surgically implanted cannula are any more ecologically relevant than air puffs. They are aversive stimuli, and as such generate largely the same activity profile in LHb as it has been previously reported in primates. Indeed, the majority of the findings are very similar to the results reported by Matsumoto 2009, though here performed here in freely mice. The main advances in my opinion, apart from the replication of the primate results in a genetically tractable model species, and in a molecularly-defined population of cells, which is certainly useful for the field, are the signal dynamics during learning, and the observation that conditioning happens over a small number of trials while not changing the response to the unconditioned stimulus.

We agree that footshocks and intra-oral delivery of quinine are not very ecologically relevant. In the revision, these statements have been toned down considerably. On the other hand, we hope that reviewer 3 can agree that it is valuable to test how LHb neurons respond to aversive stimuli targeting other sensory modalities (pain and taste). Moreover, it is important to examine how LHb neurons respond to complex, ecologically relevant aversive stimuli such as social aggression.

In addition, the social defeat paradigm is potentially interesting, and much more "ecologically relevant", but I don't think that the experimental design is appropriate to support the claims. In the current form, the authors cannot distinguish fighting-related signals from social defeat. As the only signals analyzed are during the fighting bout, it could well be that LHb neurons are signaling fighting as an aversive event, but the signal is unrelated to the outcome of the fight.

This is an extremely valuable suggestion. In the revision experiments, we separated the social defeat signals from the social fight and used appropriate controls (Figure 4). Before the test mice being repeatedly exposed to social defeat, LHb neurons did not exhibit any obvious responses when the test mouse interacted with a CD1 aggressor mouse in a mesh enclosure. Following the experience of social defeat for 10 days, LHb neurons were significantly activated when the defeated mouse interacted with an aggressive CD1 mouse in the first few bouts.

[…]. Another main comment is that it is not clear at any point in the paper how the behavior correlates with the signals. For example, for the trials shown in Figure 3E, was there freezing in response to the conditioned stimulus when there was LHb signal; or in Figure 3I, how did freezing track the reduction of LHb signals?

We agree that it would be very valuable to see how neuronal activity of LHb neurons correlates with animal behavior. In the experiments of the revision process, we further analyzed how animal locomotor activity correlates with the Ca^2+^ signals in both the conditioning phase and the extinction phase. We did not see clear relationship between the cue-evoked Ca^2+^ signals and animal locomotor speed (Author response image 2). However, this should not exclude the possibility that LHb neuronal activity is linked to fear or other aspects of aversion-related behavior. Because the current study focuses on how aversive Pavlovian conditioning shapes the response patterns of LHb neurons, we used a brief sensory cue (2 s) and short delay (2s) before US delivery. In addition, the entire recording session was completed in dark. This is rather different from standard fear freezing protocol, which often uses prolonged sensory cue (20 s) and illuminated chamber for the convenience of observing freezing responses. Nevertheless, we fully agree with reviewer 3 on the value of analyzing the relationship between LHb neuronal activity and animal behavior. It also reminds us that such experiments in future should adopt proper protocols for observing freezing responses.

**Author response image 2.** There is not clear relationship between the cue-evoked Ca^2+^ signals and animal locomotor speed. (**A**) Heatmap representation of Ca^2+^ transients during a conditioning session (n =20 trials; the same one shown in Figure 3E). (**B**) The peri-event plot of the average Ca^2+^ transient from the same mouse shown in (**A**) during the first 5 trials (black) and last 5 trials of the conditioning session. (**C**) Heatmap represents the locomotion speed of the same mouse shown in (**A**) during a conditioning session. (**D**) Walking distance during cue presentation (0-2 s; blue line) and footshock delivery (4-4.5 s; red line). Each data point represents the average of 5 consecutive trials. (E-H) The effects of omitting footshock on previously conditioned responses to the footshock-predicting cue and locomotion speed. (**E**) Heatmap representation of Ca^2+^ transients in an extinction session (30 trials), within which we repetitively presented 30 CS cues but omitted footshock. (**F**) Mean Ca^2+^ transients in one extinction session. (E and F) correspond to the same mouse in (A and B). (**G**) Heatmap represents the locomotor speed of the same mouse shown in (**A**) in an extinction session. (**H**) Walking distance during cue presentation (0-2 s; blue line) and footshock omission (4-4.5 s; red line). Each data point represents the average of 5 consecutive trials. The distance was measured as the accumulating pixel changes in video frames during either CS or US presentation. (In D, H) n.s., not significant; nonparametric one-way ANOVA for the difference between the first data point and those of the following trials. **DOI:** http://dx.doi.org/10.7554/eLife.23045.018

*[Editors' note: further revisions were requested prior to acceptance, as described below.]*

[…]

Reviewer #3:

The authors have taken my comments on board and made a serious effort to address them. The manuscript has benefited from editing, and the toning down of the ecological relevance of the stimuli used provides a more realistic account of the study. The new experiments on the social defeat paradigm are great. These are now appropriately controlled for, and the results on the exposure to the aggressor before and after social defeat make a clear case for an aversion signal (instead of a fighting-related signal) – the data are convincing, and the dynamics observed interesting.

I have, however, one major comment remaining. In response to my request for correlating neuronal activity with behavior, the authors reveal that they have not monitored behavior for most experiments. I had not realised this, and I am slightly perplexed: learning and Pavlovian conditioning are behavioral constructs, what evidence do the authors have that mice actually learned anything? While there is little doubt that a footshock or quinine are aversive, the question is whether the conditioning paradigms used do lead conditioning. If the authors cannot provide convincing evidence that this was the case, then the consequence in my view is that the terms learning and Pavlovian conditioning have to be removed altogether from the manuscript. The authors can state that they have used associative conditioning protocols, but they cannot refer to this as learning, and must make clear that the changes are only detectable at the neural activity level, but not at the behavioural level. The latter outcome is still potentially interesting, but it does require the ability to quantify the presence or absence or behavioral conditioning. If this is not possible, I have to say that my enthusiasm for the paper is severely mitigated (on a similar line, what exactly is the "reward probabilistic discriminative task"? One thing is to expose mice to rewards of different probability, another is to require them to perform a task contingent on their ability to estimate these probabilities. I have the impression that the authors did the former, and if so, this is not a "task").

I want to thank reviewer 3 for making this valuable suggestion. We have indeed monitored the locomotor activity of several mice performing cue-footshock conditioning (n = 5 mice) and cue-sucrose conditioning (n = 6 mice). Somehow we misunderstood his/her initial review comments on fear conditioning. Previously we analyzed the mouse locomotor activity during the cue (0-2 s after cue onset) and observed a trend of decrease. Since the purpose here is to test whether this training protocol led to behavioral change, we reanalyzed the data by examining animal locomotor activity during the anticipatory phase (0-4 s; between cue onset and US onset). Although the cue-footshock conditioning experiment was not performed according to the standard fear-freezing paradigm (in the dark with 2 s cue plus 2 s delay vs. in illuminated chamber with 20 s cue), we did observe a statistically significant change in behavior during the training process.

As shown in Figure 3—figure supplement 1, in the beginning trials mice exhibited active locomotor activity during the footshock-predicting period. Merely after 10 trials of training, their locomotor activity decreased significantly. During the extinction sessions, animal locomotor activity became gradually increased; the change reached statistical significance after about 25 extinction trials. These behavioral changes are temporally consistent with the changes in cue-evoked activity of LHb neurons (Figure 3H).

Our analysis of locomotor activity during cue-sucrose conditioning sessions similarly revealed good correlation between neuronal activity and behavioral changes (Figure 5—figure supplement 1). Initially, mouse locomotor activity increased during the cue and decreased upon sucrose delivery. The sucrose-associated decrease in locomotion became more pronounced as the conditioning continued; its timing gradually shifted closer to the cue, became statistically significant after over 100 trials, and became stabilized after about 300 trials. The establishment of this stable conditioned behavioral response to the cue indicated successful Pavlovian conditioning. Again, the change in locomotor activity during appetitive conditioning correlated well with the change in LHb neuronal responses to the sucrose-predicting cues (Figure 5; Figure 5—figure supplement 1).

Therefore, we have demonstrated that, based on changes in locomotor activity, the cuefootshock and cue-sucrose coupling led to behavioral changes. Moreover, these behavioural changes correlate well with the changes in the LHb neuronal activity during the conditioning process. Since the term “learning” is defined as the experience-dependent changes in behavior and neuronal activity, we indeed have good evidence to support that the changes in neuronal activity pattern shown in Figure 3 and Figure 5 resulted from learning (Pavlovian conditioning).
